# Causal assessment of dietary acid load and bone disease: a systematic review & meta-analysis applying Hill's epidemiologic criteria for causality

**DOI:** 10.1186/1475-2891-10-41

**Published:** 2011-04-30

**Authors:** Tanis R Fenton, Suzanne C Tough, Andrew W Lyon, Misha Eliasziw, David A Hanley

**Affiliations:** 1 Department of Community Health Sciences, University of Calgary, Calgary, AB, Canada; 2 Nutrition Services, Alberta Health Services, Calgary, AB, Canada; 3 Department of Pathology & Laboratory Medicine, University of Calgary, Calgary, AB, Canada; 4 Calgary Laboratory Services, Calgary AB, Canada; 5 Department of Medicine and Oncology, University of Calgary, Calgary, AB, Canada

## Abstract

**Background:**

Modern diets have been suggested to increase systemic acid load and net acid excretion. In response, alkaline diets and products are marketed to avoid or counteract this acid, help the body regulate its pH to prevent and cure disease. The objective of this systematic review was to evaluate causal relationships between dietary acid load and osteoporosis using Hill's criteria.

**Methods:**

Systematic review and meta-analysis. We systematically searched published literature for randomized intervention trials, prospective cohort studies, and meta-analyses of the acid-ash or acid-base diet hypothesis with bone-related outcomes, in which the diet acid load was altered, or an alkaline diet or alkaline salts were provided, to healthy human adults. Cellular mechanism studies were also systematically examined.

**Results:**

Fifty-five of 238 studies met the inclusion criteria: 22 randomized interventions, 2 meta-analyses, and 11 prospective observational studies of bone health outcomes including: urine calcium excretion, calcium balance or retention, changes of bone mineral density, or fractures, among healthy adults in which acid and/or alkaline intakes were manipulated or observed through foods or supplements; and 19 *in vitro *cell studies which examined the hypothesized mechanism. Urine calcium excretion rates were consistent with osteoporosis development; however calcium balance studies did not demonstrate loss of whole body calcium with higher net acid excretion. Several weaknesses regarding the acid-ash hypothesis were uncovered: No intervention studies provided direct evidence of osteoporosis progression (fragility fractures, or bone strength as measured using biopsy). The supporting prospective cohort studies were not controlled regarding important osteoporosis risk factors including: weight loss during follow-up, family history of osteoporosis, baseline bone mineral density, and estrogen status. No study revealed a biologic mechanism functioning at physiological pH. Finally, randomized studies did not provide evidence for an adverse role of phosphate, milk, and grain foods in osteoporosis.

**Conclusions:**

A causal association between dietary acid load and osteoporotic bone disease is not supported by evidence and there is no evidence that an alkaline diet is protective of bone health.

## Background

The concept that the modern diet produces excess acid, which causes several diseases of modern societies, and that "alkaline diets" prevent and cure these diseases are marketed to the general public across the globe. The public is being encouraged to measure their urine and/or salivary pH to assess their health status and their risk of disease [1-4]. Marketers claim that alkaline diets and related commercial products counteract acidity, help the body regulate its pH, and thus prevent disease processes including osteoporosis, cancer, and cardiovascular disease through websites, (e.g.[1-4]) flyers, magazines, direct mail marketing, and books [5-8] directed to lay audiences. A Google search of "acid ash diet" and "alkaline diet" resulted in 1.4 million and 400,000 hits respectively. As well, the acid-ash hypothesis has been broadly stated as a major modifiable risk factor for bone loss in osteoporosis in well cited scientific papers [9,10], textbooks [11], reference work [12], a government-funded workshop summary [13], and lay literature.

According to the acid-ash hypothesis, high dietary protein intakes are detrimental to bone health since protein is an important "acid generating" diet component, and structural bone mineral is dissolved to release bicarbonate to neutralize acid and avoid systemic acidosis [9,14-16]. A recent narrative review claimed: "acid-yielding diets (cereal grains and most dairy products) cause urinary calcium loss [and] accelerated skeletal calcium depletion..." [17]. Dietary protein associated increased urinary calcium has been considered confirmation of this theoretical effect [15,18-21].

Some critical reviews of the acid ash hypothesis have been undertaken with regards to bone health (in terms of the biochemistry [22-25], the role of protein [26,27], and phosphate [28], calcium balance [29], and the hypothesis in general [30]), however, to our knowledge, no systematic review has been done to assess the strength of the evidence of the acid ash hypothesis in terms of the etiology of osteoporosis.

The purpose of this systematic review was to evaluate causal relationships between the dietary acid load and osteoporosis among adults, and to assess the evidence using Hill's Criteria. The specific objectives were to examine the evidence that lowering the diet acid load alters the risk of osteoporosis progression by: a) conducting a thorough search of the literature for randomized human intervention and prospective observational studies and *in vitro *bone studies, of the dietary acid load and osteoporosis; b) performing meta-analyses of urine calcium, calcium balance, changes of bone mineral density (BMD), fractures, changes in bone strength, and bone resorption marker (BR marker); c) evaluating the prospective observational studies for osteoporosis risk factors that were controlled and not controlled in the analysis; d) reviewing the in vitro experimental findings to determine the pH at which increased bone resorption took place. Additional objectives of this study were to examine the exposures, the purported detrimental aspects of the diet acid load, for internal consistency, that is whether the food and urine estimates of the hypothesis were consistent with the whole hypothesis. We used Hill's criteria of causation [31] to assist in the assessment of causal relationships between exposures and disease [32,33], in this case, between the acid load of the modern diet and osteoporosis. Low quality trials are much more likely to demonstrate a benefit from an intervention [34-36], thus experts recommend that systematic reviews report meta-analyses restricted to trials at low risk of bias either as the primary analysis or in conjunction with less restrictive analyses [36]. Therefore we evaluated the included studies for their risks of bias, and focused the meta-analyses on high quality studies.

## Methods

The Preferred Reporting Items for Systematic Reviews and Meta-analyses (PRISMA) Statement [37] was used to guide this study.

### Definitions

Diet acid load - residual or excess hydrogen ion production post food metabolism

Net acid excretion - NAE = sum of urinary titratable acid and ammonium ion minus bicarbonate, usually measured in 24-hour urines

Osteoporosis - "a skeletal disorder characterized by compromised bone strength predisposing a person to an increased risk of fracture. Bone strength primarily reflects the integration of bone density and bone quality" [38].

## Eligibility Criteria

### Inclusion criteria

1) Randomized intervention, 2) prospective observational (cohort) human studies, and 3) *in vitro *animal studies of the mechanism of the acid-ash hypothesis among adults. Only studies of adults were included to avoid the potential confounding of growth and the variable timing of growth spurts. Random intervention studies were included if a) acid-base intake was manipulated through supplemental salts (such as potassium bicarbonate) or through foods to decrease the diet acid load (referred to in this text as the "alkaline diet") for, b) at least 24-hours to avoid variability due to diurnal variation, and c) outcomes related to bone health or osteoporosis (bone strength as measured with biopsy, fractures, change of BMD, calcium balance, bone resorption markers, urine calcium) were evaluated. Calcium balance studies were only included if the recommendations of the Institute of Medicine [39] for this type of study were followed, including control of calcium intake for at least 7 days prior to the measurement of outcomes, provision and precise measurement of the food to the subjects, and chemical analysis of calcium in the food.

### Exclusion criteria

1) studies with no original research (narrative reviews, editorials), 2) non-human studies (except for the *in vitro *mechanism studies), 3) studies with no control group, 4) non-prospective studies (cross-sectional or ecologic design studies). Randomized studies were favored since randomization is an indicator of rigor that reduces the probability of bias or confounding by known and unknown variables, with "numerous advantages and no disadvantages" [40]. This requirement for randomization was also applied to cross-over trials, since randomization to the order of treatments is important since without random allocation, the first treatment could influence the second period results [41].

Since the intent of this systematic review was to summarize the evidence regarding the potential for manipulation of the diet acid load as a therapy or prevention of osteoporosis for apparently healthy subjects, trials were included only if the nutritional intakes used could be recommended safely, not less than the Dietary Reference Intakes or higher than the Tolerable Upper Limits [39,42]. Studies were excluded if the subjects had conditions such as renal diseases, or were in states (such as asphyxia, diabetic keto-acidosis, drug abuse, poisoning, calorie restriction, or decreased ambulation) which could alter the effect of the exposure on the outcome.

## Literature Search

In an attempt to find all published literature on the topic, studies relating to the acid-ash diet hypothesis and bone health were identified through computerized searches using, but not limited to, the medical subject headings and textwords, first: *acid, alkaline, acid-ash, acid-base, modern, western, diet, calcium, phosphate, acid-base equilibrium, acid excretion, net acid excretion, bone or bones, osteoporosis, urine, balance/retention, biopsy, fracture(s), bone mineral*, and *bone mineral density*. Second, to find studies that have examined proposed mechanisms for the hypothesis, we used the terms *hydrogen-Ion concentration, cells (cultured)*, and *mechanism*. Databases searched included Medline back to 1966 (PubMed), Cochrane Database of Systematic Reviews, CINAHL back to 1982, EMBASE back to 1980, and the Cochrane Controlled Trials Register, up to August 2010. A Librarian (DL) was consulted regarding the literature search. In an effort to include all available studies, reference lists were reviewed for additional relevant articles. The literature search was not limited to English language articles.

Article titles were examined for potential fit to the inclusion criteria by one reviewer (TRF). When the title was not clear regarding the potential fit, the abstract was reviewed; when the abstract was not clear regarding whether the study fit the inclusion criteria, the paper was reviewed. Authors were contacted for additional information. Two authors (SCT & TRF) independently rated the randomized studies for their risk of bias using the Cochrane Risk of Bias Tool [43]; two (AWL & TRF) extracted the BR marker data; and two (ME & DAH) extracted the potential confounders controlled in the cohort studies. Differences of opinion were resolved by discussion to achieve consensus.

After the data, including exposures and outcomes, was extracted and described in tables, the risk of bias of the randomized studies was assessed using the Cochrane Risk of Bias Tool [43], for the randomized studies of the acid ash hypothesis by SCT and TRF.

## Meta-Analysis Methods

To address the questions of what evidence supports the acid-ash hypothesis for the role of net acid excretion (NAE) and phosphate in urinary calcium excretion and calcium balance, we used the highest quality of evidence available, meta-analyses of random control trials (RCTs) or random cross-over studies (RCO). When meta-analyses were not found that fit the inclusion criteria and randomized trials were found that did meet the criteria, then meta-analyses were performed. When the exposure was a continuous measure, then linear regression analyses, weighted for sample size, were used to combine the results from the included studies to examine the effect of NAE and dietary phosphate on urinary calcium excretion and calcium balance.

To examine the effect of an alkaline treatment or a reduced "acid" diet load on the changes of resorptive bone resorption markers (BR markers) (i.e. serum C-telopeptide (CTX), urine N-telopeptide, and urine deoxypyridinoline crosslinks) using meta-analysis techniques, the exposures were considered as alkaline versus control, and standardized mean differences were calculated using fixed and/or random effects models, with Cochrane RevMan5 (Version 5.0. Copenhagen, The Cochrane Collaboration, 2008). If the p-value for heterogeneity was between 0.05 and 0.5, then a random effects model was used [44]. The BR marker changes from baseline were used when baseline values were available in the RCTs, or the differences between the groups if baseline values were not available. Urine CTX was not included since it is considered less valid than serum CTX due to higher biological variation [45]. Then a second meta-analysis was performed as a sensitivity analysis, on the BR markers measured in a fasting state and at the same time of day as recommended to decrease measurement errors [46,47]. A difference greater than 30% for serum markers or 50-60% for urine markers were considered clinically important [46,47].

The *in-vitro *cell culture studies of bone demineralization at varying pH were examined to determine the pH of testing and to summarize the effects revealed within the physiological pH range. 

Prospective observational studies of the acid-ash hypothesis were examined for whether they supported or did not support the hypothesis, as well as which osteoporosis risk factors [48-52] were controlled for in the analysis.

Hill's criteria of causation [31] (Table 1) was used to evaluate the possibility of causation by the acid load of the modern diet on the etiology or potentiation of osteoporosis. Hill's criteria include: whether the exposure precedes the disease in time (Temporality), whether a dose-response or Biological Gradient relationship exists, the Strength of the evidence, whether the concept is Biologically Plausible, whether the evidence has Consistent findings between the studies of various designs, and whether actual Experiments have been done to determine whether altered exposure results in changes in disease frequency [31].

**Table 1:** HILL'S CRITERIA OF CAUSATION

Criteria	Description
TEMPORALITY	An exposure must be measured prior the disease, for it to be clear which variable might be the cause and which variable might be the result.

STRENGTH	This criterion requires that the putative cause of an illness be of sufficient strength of association to cause disease.

BIOLOGICAL GRADIENT	This criterion requires that when the dose of an exposure is increased, the risk of the outcome should also increase

PLAUSIBILITY	This criterion requires that a theory fit with current biological knowledge.

CONSISTENCY	This criterion requires consistent evidence from a variety of study designs to support a causal relationship

EXPERIMENT	This criterion requires that actual experiments be conducted to determine whether the frequency of a disease is altered by an exposure.

## Results

### Description of Studies

**Figure 1:**
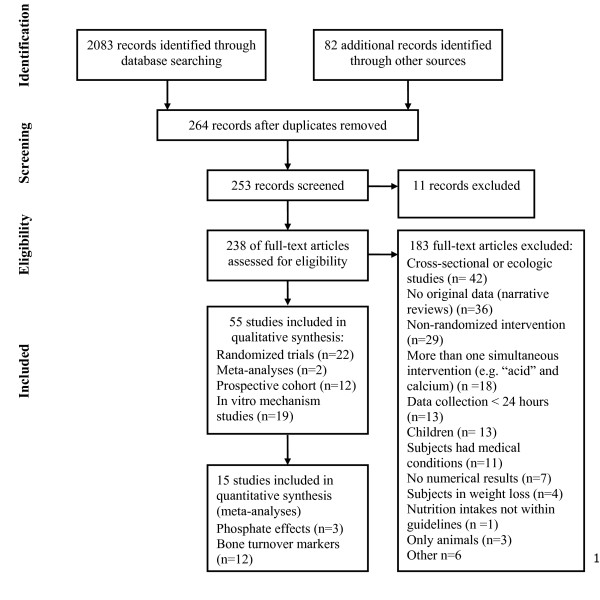
**Flow diagram of studies identified, excluded and included in the systematic review**.

Out of 2165 references identified, fifty-five of 238 studies of the acid-ash hypothesis met the inclusion criteria (Figure 1). Twenty-two randomized intervention studies [14,20,53-72] (Table 2), two meta-analyses [27,29], and 12 prospective observational cohort studies (Table 3) [73-84] of the hypothesis met the inclusion criteria for at least one part of the study. Nineteen in-vitro animal bone mechanism studies were located [85-103]. The outcomes in the randomized intervention studies included: two examined changes of bone mineral density (BMD) [65,68], 15 examined calcium absorption and/or balance [14,20,53,55-62,67,69-71], five examined changes of urine calcium without calcium balance [54,63,64,66,72], 12 examined changes in BR markers [14,57,59-61,65,67-72], and none reported fractures or bone strength. The 12 prospective observational (cohort) studies examined fractures [73,74,80,81] and/or changes of BMD [75-79,82-84].

**Table 2:** Randomized intervention Human Studies that Met the Inclusion Criteria

					Cochrane Risk of Bias Assessment			
					
Study	Design	Exposures	Exposure quantified	Outcomes	Sequence generation	Allocation concealment	Incomplete outcome data	Selective outcome reporting
Patton [53]	RCO	Phosphate salt	mg P	Calcium balance	low	High	low	low
Breslau [20]	RCO	Protein foods	NAE	Urine calcium & absorption	low	High	low	Low
Whybro* [54]	RCO	Phosphate salt	mmol P	Urine calcium	part 1 = low	High	low	low
Dahl [55]	RCO	Lentils	NAE	Calcium balance	low	High	low	low
Kerstetter [56]	RCO	Amount of protein	g protein	Urine calcium, absorption & BTM	low	High	low	low
Buclin [14]	RCO	Acid diet	No	Urine calcium & BTM	low	High	low	low
Roughead [57]	RCO	Amount of protein	NAE	Calcium balance & BTM	low	High	low	low
Dawson-Hughes [58]	RCT	Amount of protein	g protein	Urine calcium & BTM	low	High	low	low
Roughead [59]	RCO	Meat/soy	NAE	Calcium balance & BTM	low	High	low	low
Sakhaee [60]	RCO	K+citrate	K+citrate	Urine calcium, absorption & BTM	low	High	low	low
Spence [61]	RCO	Soy vs. milk protein	NAE	Calcium balance & BTM	low	High	low	low
Kerstetter [62]	RCO	Amount of protein	NAE	Calcium balance	low	High	low	low
Kemi [66]	RCO	Phosphate salt	mg P	Urine calcium	low	High	low	low
Kerstetter [67]	RCO	Amount & type of protein	NAE	Calcium balance & BTM	low	High	low	low
Hunt [69]	RCO	Protein	g protein, mg Ca	Calcium balance & BTM	low	High	low	low
Ceglia* [70]	RCT	K+bicarbonate	NAE	Urine calcium and absorption	low	High	low	low
Dawson-Hughes [70]	RCT	K+bicarbonate	NAE	Urine calcium & BTM	low	High	low	low
Frassetto [63]	RCT	K+bicarbonate	K+bicarbonate	Urine calcium	low	High	low	low
Gettman [64]	RCO	Cranberry juice	NAE	Urine calcium	low	High	low	low
Karp [72]	RCO	K+citrate	K+citrate	Urine calcium & BTM	low	High	low	low
Jehle [65]	RCT	K+citrate	NAE	BMD & BTM	low	High	low	High
MacDonald [79]	RCT	K+citrate/fruit & veg	NEAP	BMD & BTM	low	low	low	low

BMD = bone mineral density; BTM = bone turnover markers; ca = calcium, K+ = potassium; mg = milligram; mmol = millimole; NAE = Net acid excretion; NEAP =, net endogenous acid production; P = phosphate; RCO = random cross-over study; RCT = random control trial; veg = vegetables; * = only the randomized portions of this study fit the inclusion criteria

**Table 3:** Prospective Observational Studies that met the Inclusion Criteria

Study	Year	Population	Exposures	Outcomes	Results	Potential confounders controlled or stratified	Potential confounders not controlled
Feskanich	1996	Women 35 to 59 years	Protein intake	Fractures	Protein intake was associated with increased risk of forearm fracture; no association between protein intake and hip fractures.	Age, BMI, change of BMI, estrogen status, smoking, energy intake, physical activity, calcium, potassium, and vitamin D intakes.	Family history of osteoporosis, baseline BMD

Munger	1999	Postmenopausal women	Protein intake	Hip fractures	Protein intake was associated with lower hip fracture risk.	Age, body size, parity, smoking, alcohol intake, estrogen use, physical activity	Weight loss during follow-up, family history of osteoporosis, baseline BMD, vitamin D status, calcium intake

Tucker	2001	Adults 69 to 97 years	Fruit & vegetable nutrients, & protein	Change of BMD	Potassium, fruit & vegetable intakes among men were associated with less BMD loss. Protein intakes were associated with less BMD loss.	Energy intake, age, sex, weight, BMI, smoking, caffeine, alcohol intake, physical activity, calcium intake, calcium and/or vitamin D supplements, season, current estrogen use.	Weight loss during follow-up, family history of osteoporosis, baseline BMD

Promislow	2002	Adults 55 to 92 years	Protein intake	Change of BMD	Protein intake was associated with increased BMD over 4 years.	Energy intake, calcium intake, diabetes, number of years postmenopausal, exercise, smoking, alcohol, thiazides, thyroid hormones, steroids, and estrogen, body weight change	Family history of osteoporosis, baseline BMD

Kaptoge	2003	Adults 67 to 79 years	Fruit, vegetables, vitamin C	Change of BMD	No associations between nutrients and BMD loss. In women, vitamin C was associated with less BMD loss. No associations for fruit and vegetable intakes.	Sex, age, BMI, weight change, physical activity, smoking, family history, energy intake.	Baseline BMD, estrogen status, vitamin D status, calcium intake

Rapuri	2003	Women 65 to 77 years	Protein intake	Change of BMD	No association between protein intake and the rate of bone loss.	Age, BMI, intakes of calcium, energy, fiber, vitamin D status, and alcohol, smoking, physical activity.	Weight loss during follow-up, baseline BMD, family history of osteoporosis

MacDonald	2004	Premenopausal women	Fruit & vegetables nutrients	Change of BMD	Among menstruating and perimenopausal women, intakes of vitamin C and magnesium, but not potassium, were associated with change of BMD.	Age, weight, change in weight, height, smoking, physical activity, socioeconomic status, baseline BMD.	Family history of osteoporosis, calcium intake, vitamin D status

Dargent-Molina	2008	Postmenopausal women	Protein & diet acid load	Fractures	No overall association between protein intake and acid excretion with fracture risk; in the lowest calcium intake quartile, protein intake was associated with fracture risk	Age, BMI, physical activity, parity, maternal history of hip fracture, hormonal therapy, smoking, alcohol, energy intake.	Weight loss during follow-up, baseline BMD, vitamin D status.

Thorpe	2008	Peri- and Postmenopausal women	Protein	Wrist fractures	Protein intake was associated with lower risk of wrist fracture, for both vegetable and meat protein.	Age, height, weight, BMI, education, any fracture since age 35, parity, smoking, alcohol use, diabetes mellitus, rheumatoid arthritis, physical activity, years since menopause.	Estrogen status, calcium intake

Pedone	2009	Women 60 to 96 years	Potential renal acid load	Change of BMD	Protein intake was associated with a lower loss of BMD.	Physical activity, energy intake, renal function, vitamin D status, estrogen status, baseline BMD.	Weight loss during follow-up, family history of osteoporosis, calcium intake.

Beasley	2010	Women 14 to 40 years	Protein intake	Change of BMD	No association between protein intake and change of BMD.	Age, race-ethnicity, age of menarche, time since menarche, family history of fracture, BMI, physical activity score, calories, dietary calcium, phosphorous, dietary vitamin D, magnesium, fluoride, alcohol, smoking, contraceptive use, prior pregnancy, and education	

Fenton	2010	Adults 25 years+	Urine pH, urine potassium, sodium, calcium, magnesium, phosphate, sulfate, chloride, and acid excretion, controlled for urine creatinine	Change of BMD and fractures	No associations between urine pH or acid excretion and either the incidence of fractures or change of BMD	Age, gender, family history of osteoporosis, BMI, change in BMI, baseline BMD, estrogen status, kidney disease, smoking, thiazide diuretics, bisphosphonates, physical activity, calcium intake, and vitamin D status, urine creatinine,.	

* BMD = bone mineral density; BMI = body mass index

Using the Cochrane Risk of Bias Tool [43] to assess the risk of bias of the randomized studies, all studies were assessed as having a low risk of bias in terms of blinding [43] since the outcomes were objective measures. All of the studies were ranked by two authors as having a low risk of bias for sequence generation and complete data accounted for (Table 2). Only one study [65] was rated as having a high risk of selective outcome reporting bias. In terms of allocation concealment, only one study [68] demonstrated adequate procedures to avoid this risk of bias. The identified risks of bias ranged from zero to two risks of bias, with only one study rated as free of potential bias [68] (Table 2).

To quantify the acid or alkali load exposure, in the randomized trials, 12 of the 22 reported a measure of net acid excretion (NAE), a urinary measure of acid excretion, and one reported net endogenous acid production, an estimation of acid excretion based on food intake and body size. Three of the studies provided an alkaline or acid intervention in terms of bicarbonate salt, three others with a phosphate salt, and the final three studies altered protein intake, considered by the researchers to be an acid exposure.

Some acid-ash hypothesis studies, some that have been frequently quoted in the lay literature, were ineligible for inclusion because they were not randomized [9,15,18,21,104-131], had unclear methodology [132], and randomization was questionable due to high baseline differences between the two groups [133]. One well quoted study incorporated a salt intake dose of 225 mmol/day (5.2 gram/day) for both the potassium citrate and placebo groups [134] that was more than twice the Tolerable Upper Limit of 2.3 gram/day [12]. Two studies used a protein intake that were less than the recommended Dietary Reference Intake in one arm of each study, so that arm was not included [56,70]. Two meta-analyses of intervention studies of the hypothesis were not included since they did not limit inclusion to randomized trials [28,135]. Numerous studies were cross-sectional observation [19,73,136-173] or ecologic studies [174,175] (which lack the ability to assess temporality) did not meet the inclusion criteria. One case-control study was located [176] was not included because the design is not well suited to assessing causality (eg recall bias, retrospective data collection) [41].

Other reasons for excluding studies were: No numerical results presented and no response to a written request [177,178]; more than one simultaneous intervention [179-190], a simultaneous co-intervention of change of "acid" as well as other potentially bone influencing nutrients including calcium, sodium, potassium, magnesium, and/or phosphate [130,191-194], hypothesis generating studies that lack a no-intervention control group [195-197], time periods were shorter than 24 hours [198-210], did not include outcomes required in the inclusion criteria [211]; all of the subjects had a chronic medical condition [124,212-220], were on medications [221], or were in a state of weight loss [222-225], only included children [106-108,137,138,141-143,145,149,155,166,202], or only included animals [226-228]. Two studies [229,230] were subsets of included studies [56,75]. The search also located numerous narrative review articles on the acid-ash hypothesis [10,16,22-26,132,231-258] which did not qualify for inclusion in this systematic review. No foreign language articles [124,130,231] met the inclusion criteria.

#### Urine calcium and calcium balance studies - Acid excretion

Based on a meta-analysis that met the inclusion criteria of this systematic review, the estimated excess calciuria from the diet acid load is 66 mg/day (Confidence interval = 60 to 71 mg/day) (1.6 mmol/day, confidence interval = 1.5 to 1.8 mmol/day), based on diets designed to represent the modern acid-generating diet [135]. If this calcium loss estimated from short term studies were extrapolated over time, without adaption, a continuous loss of 66 mg/day would lead to 24 grams/year or 480 grams over 20 years.

**Figure 2:**
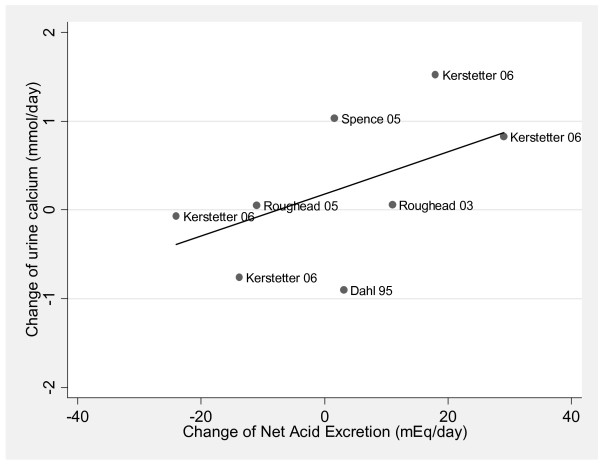
**The relationship between change in NAE and change in urinary calcium, limited to randomized studies that followed the Institute of Medicines' guidelines for calcium metabolism studies (R2 = 0.406; p < 0.0001)**. This material is reproduced with permission of John Wiley & Sons, Inc. from Fenton et al. J Bone Miner Res 2009;24:1835-1840.

**Figure 3:**
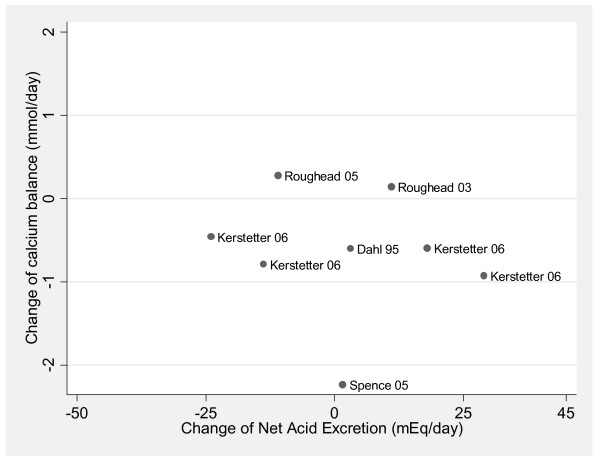
**No relationship between change in NAE and change in calcium balance, analysis limited to randomized studies that followed the Institute of Medicines' guidelines for calcium metabolism studies (R2 = 0.003; p = 0.38)**. This material is reproduced with permission of John Wiley & Sons, Inc. from Fenton et al. J Bone Miner Res 2009;24:1835-1840.

Among the studies included in this meta-analysis [135], investigators exposed subjects to a wide range of acid or base treatments, between a decrease NAE of 57 [70] to an increase of 69 [62] milliequivalent/day. As NAE is increased, the excretion of calcium in the urine also increased. For every milliequivalent increase of NAE, urine calcium increased by 0.03 mmol/day (95% confidence interval (CI) = 0.023 to 0.035, p < 0.0001) (n = 133) (Figure 2) [29]. However, a meta-analysis of five randomized calcium balance studies with superior methodology revealed no evidence that diet changes that raise NAE lowers calcium balance (n = 77, p = 0.38) (Figure 3) [29].

A randomized calcium balance study was published since this meta-analysis [29] was completed. This randomized trial of two levels of protein intake on calcium metabolism by Hunt et al. [69] found that higher protein intakes were not detrimental to calcium retention since higher protein increased calcium absorption in similar quantity to the rise in urine calcium, even when calcium intakes were low [69].

#### Urine calcium and calcium balance studies - Phosphate Studies

**Figure 4:**
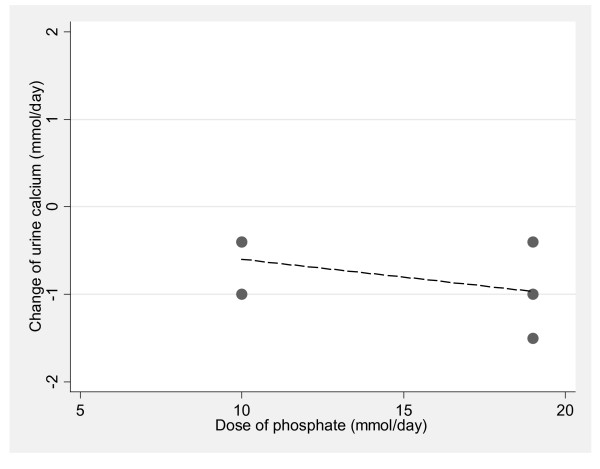
**The relationship between phosphate supplementation and change urine calcium, limited to randomized studies that followed the Institute of Medicines' guidelines for calcium metabolism studies (R^2 ^= 0.185 p < 0.001)**.

**Figure 5:**
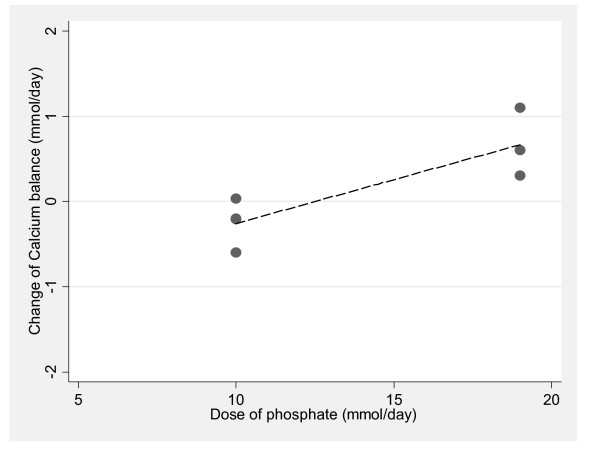
**The relationship between phosphate supplementation and change calcium balance, limited to randomized studies that followed the Institute of Medicines' guidelines for calcium metabolism studies (R^2 ^= 0.704, p < 0.001)**.

*Phosphate*. In a meta-analysis of the influence of phosphate supplements on calcium metabolism, limited to randomized studies that followed the recommendations of the Institute of Medicine for calcium balance studies [39], only one study met the inclusion criteria [53]. This cross-over study randomized young adult women to two phosphate doses (300 mg = 10 mmol and 600 mg = 20 mmol) stratified by calcium intakes of 344 mg (9 mmol) (basal diet) or with calcium supplements of 600 (15 mmol) or 1200 mg (30 mmol) per day. The regression analysis of the effect of phosphate on urine calcium revealed a statistically significant linear relationship (Figure 4). For every mmol increase in phosphate supplement, urine calcium decreased by 0.04 mmol/day (95% CI = -0.06 to -0.02, p < 0.001). For calcium balance, the relationship was in the opposite direction (Figure 5). For every mmol increase in phosphate supplement, calcium balance increased by 0.10 mmol/day (95% CI = 0.09 to 0.12, p < 0.001).

#### Protein

Regarding the role of protein and bone demineralization, recent randomized cross-over studies of the amount [57,62,67,69] and type (animal versus vegetable) [59] of protein, and a meta-analyses of superior methodology randomized cross-over studies of protein intakes on calcium balance [29] demonstrated that higher protein intakes and animal protein were not detrimental to calcium retention.

#### Prospective observational studies

Prospective observational studies measure an exposure prior to the outcome and therefore met the inclusion criterion of Temporality, but these studies are not randomized since the subjects chose their own lifestyles and the investigators only observe the outcomes. Twelve prospective observational cohort studies examined associations between either fruit and vegetable intakes, related nutrients [75,77,79], protein intakes [73-76,78,80-82,84], or urine measures of acid excretion [83] with changes in BMD [75-79,82-84] and/or fractures [73,74,80,81,83] as the outcomes (Table 3). Five of these studies had some results that supported the acid-ash hypothesis [73,75,77,79,81]; while the results of seven studies did not support the hypothesis [74,76,80-84]. Each of the prospective cohort studies that supported the hypothesis also had some findings that did not support the hypothesis [73,75,77,79,81].

#### In vitro studies of animal bone

*In vitro *studies of animal bone demonstrated higher rates of bone demineralization when exposed to low pH below the physiological range (≤ 7.3) and calcium release from bone [85], activation of osteoclasts [96], and enzyme activities [88]. None of the reports mentioned conducting tests within the physiological range [85-89,91,94-97,99,100,102] or at any pH greater than 7.2 [89,94,96].

#### Bone outcomes used in the experimental studies of the acid ash hypothesis

No experimental studies were found that examined bone strength (which is considered the best outcome measures of osteoporosis, that is fragility fractures or bone strength as measured from bone biopsy samples [259,260]).

#### Changes in Bone Mineral Density

Two RCTs used changes of BMD as the outcome measure in post menopausal women with opposite BMD findings [65,68]. Both of these studies examined the effect of potassium citrate on the change of BMD over one [65] or two [68] years, and one also examined the effect of increased fruit and vegetable intakes [68].

The studies differed in quality by two important risk of Bias [43]: concealment of allocation and selective outcome reporting (Table 2). The study by MacDonald et al concealed their subject allocation to the groups [68], while the other study by Jehle et al did not [65]. The Jehle et al paper does not report their changes of BMD or BR markers numerically "so that they could be included in a meta-analysis" [43]. MacDonald et al found no effect of the potassium citrate, or increased fruit and vegetable intakes on BMD over time [68], while the Jehle et al study reported an increase in BMD at femoral neck (*P* < 0.001), and at total hip (*P *< 0.001) in the potassium citrate group.

#### Bone resorption markers (BR markers)

**Figure 6:**
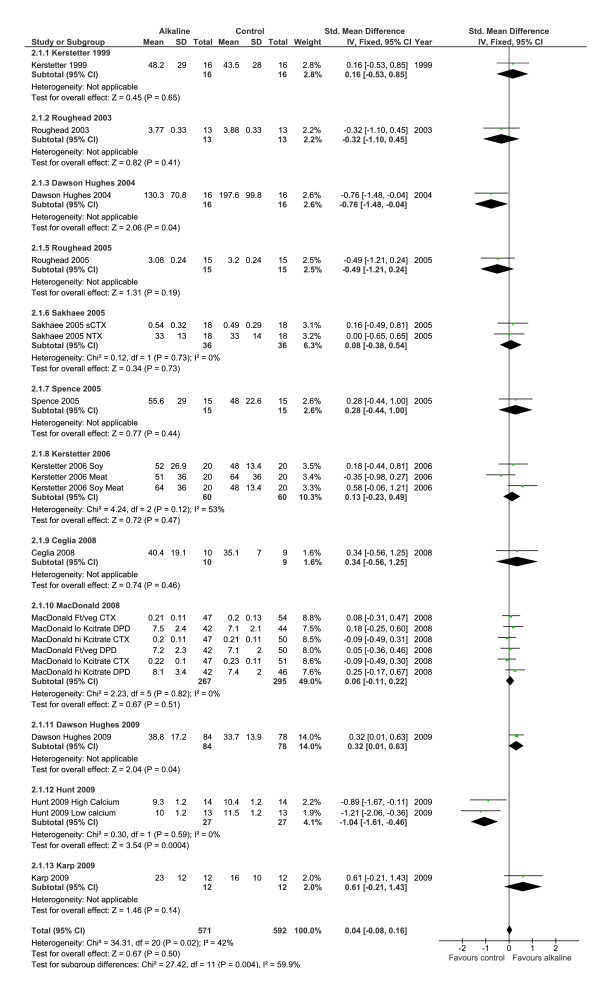
**Changes of bone resorption markers in response to a more alkaline diet**. The results were heterogeneous (p = 0.02), therefore it was not considered valid to combine them and examine the test for overall effect.

**Figure 7:**
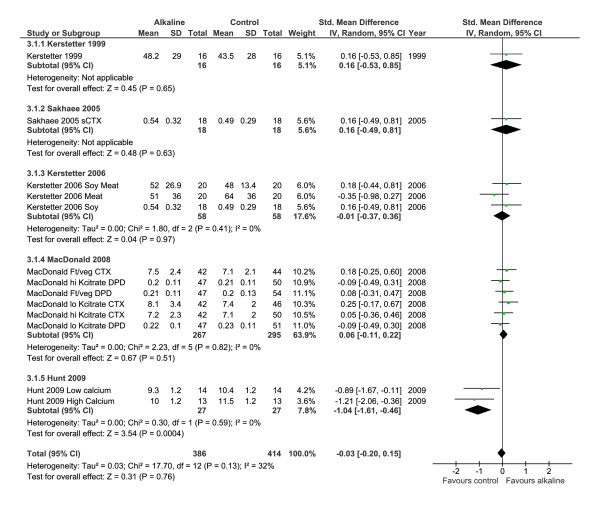
**Changes of bone resorption markers in response to a more alkaline diet, measured while subjects were fasting**. The relationship between interventions to alter the diet acid load on bone resorption markers was not significant effect (p-value = 0.91).

When all of the individual studies of alkaline treatments or reduced "acid" diet loads on the changes of BR markers were combined in a meta-analysis, the individual study findings were significantly heterogeneous (p = 0.02) (Table 4) (Figure 6), thus the results from all the studies should not be combined into a summary effect estimate [43]. In the sensitivity analysis, limited to samples collected under fasting conditions (Figure 7), the study results were sufficiently homogenous that the meta-analysis results could be considered (test of heterogeneity p-value = 0.13, non-significant), and a random effect model was used [44]. The estimated  summary effect of an alkaline intake on BR markers taken under fasting conditions revealed no overall effect (p = 0.76) in meta-analysis (Figure 7).

**Table 4:** Change in Bone Resorption Markers in Response to a More Alkaline Diet

1st Author	year	n	Subjects	Comparison	Design	Change NAE	Marker	Fasting	Control	Alkaline	Percent change of marker
Kerstetter	1999	16	Women 20 - 40 years	High vs medium pro	RCO		NTX/Cr	yes	48.2 (29)	43.5 (28)	-10*
Roughead	2003	13	Postmeno women	High to low meat	RCO	-19	NTX/Cr	no	3.77 (0.33)*	3.88 (0.33)*	11
Dawson- Hughes	2004	32	Adults > 50 yrs	High vs low pro	RCT		NTX/Cr	no	130 (71)	198 (100)	52*
Roughead	2005	15	Postmeno women	Milk to soy pro	RCO	-11	NTX/Cr	no	3.08 (0.24)*	3.20 (0.24)*	15
Sakhaee	2005	18	Postmeno women	Kcitrate	RCO	0	NTx/Cr	no	33 (13)	33 (14)	0.0
Sakhaee	2005	18	Postmeno women	Kcitrate	RCO	0	sCTX	yes	0.54 (0.32)	0.49 (0.29)	-9.3
Spence	2005	15	Postmeno women	Milk to soy pro	RCO	-2	NTX/Cr	no	55.6 (29.0)	48 (22.6)	-14
Kerstetter	2006	20	Women	Amt of soy	RCO	-29	NTx/Cr	yes	52 (27)	48 (13)	-7.7
Kerstetter	2006	20	Women	Soy versus meat	RCO	-24	NTx/Cr	yes	64 (36)	48 (13)	-25
Kerstetter	2006	20	Women	Amt of meat	RCO	-18	NTx/Cr	yes	51 (36)	64 (36)	25
Ceglia	2008	19	Adults > 50 yrs	KHCO3 (high pro)	RCT	-57	NTX/Cr	no	40.4 (19.1)	35.1 (7.0)	-13
MacDonald	2008	46	Postmeno women	Kcitrate (high)	RCT		DPD/Cr	yes	8.1 (3.4)	7.4 (2.0)	-8.6
MacDonald	2008	44	Postmeno women	Kcitrate (low)	RCT		DPD/Cr	yes	7.5 (2.4)	7.1 (2.1)	-5.3
MacDonald	2008	50	Postmeno women	Ft & veg	RCT		DPD/Cr	yes	7.2 (2.3)	7.1 (2.0)	-1.4
MacDonald	2008	50	Postmeno women	Kcitrate (high)	RCT		sCTX	no	0.21 (0.11)	0.20 (0.11)	-4.3
MacDonald	2008	51	Postmeno women	Kcitrate (low)	RCT		sCTX	no	0.23 (0.11)	0.22 (0.10)	-4.3
MacDonald	2008	54	Postmeno women	Ft & veg	RCT		sCTX	no	0.20 (0.13)	0.21 (0.11)	5.0
Dawson- Hughes	2009	162	Adults > 50 yrs	K or Na HCO3	RCT	-35	NTX/Cr	no	38.8 (17.2)	33.7 (13.9)	-13
Hunt	2009	13	Postmeno women	high to low pro (low Ca)	RCO	-24	DPD/Cr	yes	2.3 (0.2)*	2.4 (0.2)*	15*
Hunt	2009	14	Postmeno women	high to low pro (High Ca)	RCO	-22	DPD/Cr	yes	2.2 (0.2)*	2.3 (0.2)*	12*
Karp	2009	12	Women 20 - 30 years	Kcitrate	RCO		NTX/Cr	no	23 (12)	16 (10)*	-28*

* p < 0.05

DPD/Cr = urine deoxypyridinoline to creatinine ratio; NTX/Cr = urine N-telopeptide to creatinine ratio; RCT = random control trial; RCO = random cross-over study; pro = protein, sCTX = serum C-telopeptide, * ln transformed data

In terms of percent difference between study interventions in the individual studies, only one study reported a clinically important difference [46,47], a 52% decrease of N-telopeptide, collected under fasting conditions, in response to protein supplementation [58]. The results which were the opposite direction to that which the hypothesis would predict. Two individual studies found individual statistically significant decreases in their subjects' BR marker, both these changes in response to higher protein intakes [58,69] were in the opposite direction to that which the hypothesis would predict, and one in the same direction [56]. The only statistically significant result that was in the direction the acid ash hypothesis would predict was in a study of bicarbonate supplementation [71]; this study was not included in the sensitivity analysis since the BR marker were not collected during fasting.

#### Potassium

None of the seven randomized intervention studies that altered potassium intake (using potassium bicarbonate) [58,60,65,68,70-72] followed the recommendations for calcium balance studies, so they were not included in the systematic review of calcium balance. In terms of BR markers, in response to potassium bicarbonate or citrate, BR markers decreased by nine [60,68] to 28 [72] percent. One prospective observational study, by Tucker at al., found an association between potassium intakes and fruit and vegetable intakes with less BMD loss [75]. The study by Tucker et al did not control for weight loss during follow-up, family history of osteoporosis, baseline BMD [75]. Additionally, this finding of association between potassium intakes and BMD loss by Tucker et al. was not substantiated by others [77,79,83]. Further, the study by Fenton et al found no association between urine potassium and either BMD loss or fractures [83].

#### Protein and calcium interaction

Regarding the assertion that that calcium and protein work in an interaction, such that protein is more detrimental when calcium intakes are low was supported by one observational study [81], but not supported by one randomized trial [69]. Dargent-Molina reported that among subjects in the lowest calcium intake quartile, protein intake was associated with higher fracture risk [81]. This study did not control for weight loss during follow-up, baseline BMD, and vitamin D status (Table 3) [81]. A randomized trial that examined the effect of two levels of calcium intake (700 vs. 1500 mg/day) with two levels of protein intake (60 vs. 110 g/day) in a controlled feeding study of postmenopausal women found that the higher protein intakes increased calcium retention from the low-calcium but not the high-calcium diet, which does not support the hypothesis [69]. The protein effects on urine calcium were independent of calcium intake [69].

## Discussion of The Hill's Criteria Assessment

### Hill's Criterion #1: Temporality

An *a priori *criterion was to include only prospective studies (Table 1), therefore, cross-sectional or ecologic design studies were not included in this systematic review (Table 5). Knowing that the exposure preceded a disease is considered "the only absolutely essential criterion" of causation [41].

**Table 5:** Summary Table of the Evaluation of the Acid-ash Hypothesis using Hill's Criteria

Hill's criterion	Is criterion met?	Reason
Temporality	Yes, by inclusion criteria	Papers were included only if this Temporality criterion was met, that is the exposure preceded the outcome.

Strength	Yes	Estimates of calcium loss in the urine are of sufficient magnitude to explain the progression of osteoporosis, while calcium balance studies do not show support of the acid ash hypothesis.
	No	

Biological Gradient or Dose-response	No	While urine calcium changes in response to changes in net acid excretion, calcium balance does not. Calcium balance is a better measure of whole body calcium metabolism than urine calcium.

Biologically Plausible	No	No defined mechanism that could take place at physiological pH.
	No	Problems with the hypothesis due to the incongruent roles of phosphate, sodium, and protein with bone, and lack of support for the role of potassium.

Consistency	No	The prospective observational cohort studies have not consistently controlled for the key osteoporosis risk factors, putting their findings into question.
	No	The estimated effects of protein, milk and grain foods are not supported by evidence.
	No	The measurement of urinary acid excretion is not a precise science and measurements may be inaccurate.

Experiments	No	The outcome measures used to date in experimental studies are only surrogate measures or correlates of bone strength. The majority of experimental evidence supporting the acid-ash hypothesis is from studies that used urine calcium and/or bone resorption markers as the outcomes, which are surrogate measures of bone strength.
	No	The RCT that assessed changes of BMD with the lower risk of bias did not support the hypothesis. Therefore, the experimental evidence does not support the hypothesis
	No	Meta-analyses of bone resorption markers in response to changes in acid and alkali loads did not support the hypothesis whether all of the study results were combined or only studies that followed recommendations for bone markers were assessed.

### Hill's Criterion #2: Strength

In terms of urine calcium, the magnitude of excess calciuria induced by the modern diet is sufficient to lead to the development of osteoporosis, or equivalent to an estimated loss of 480 grams over 20 years, almost half of the skeleton calcium [261]; a substantial loss of bone mineral, which would be considered rapidly progressing osteoporosis. Therefore, the calciuria associated with the modern diet is sufficient in quantity that it could explain the progression of osteoporosis, if the excess urine calcium is only derived from bone. In contrast, the evidence from studies of the diet acid load and calcium balance does not support the acid ash hypothesis [29]. Consequently, Hills criteria of strength of association is met if urine calcium is considered as the outcome, but not if whole body calcium balance is considered (Table 5).

### Hill's Criterion #3: Biological Gradient

The change in urine calcium may not represent a change in body calcium balance, and reporting the urine calcium without other measures of calcium flux (absorption [62], intestinal secretion [61], or fecal losses) may interfere with accurate interpretation. Methodologically superior calcium balance studies, which provide a more accurate assessment of whole body calcium metabolism compared to urine calcium, do not support the acid ash hypothesis [29].

Hill's criterion of causation regarding a Biological Gradient is substantiated by the evidence regarding urine calcium, but the evidence regarding calcium balance, the superior measure of whole body calcium metabolism does not support the acid ash hypothesis (Table 5).

### Hill's Criterion #4: Plausibility

Regarding Hill's plausibility criterion that a theory fit with current biological knowledge, the mechanism for diet acid load induced mineral resorption at the bone is not well described. Some researchers assert that bone is dissolved, releasing skeletal calcium and bicarbonate to neutralize the systemic acidemia [9,113,127,133,134,221]. Others hypothesize that the effect occurs at the kidney: calcium is lost in the urine as urinary bicarbonate is reabsorbed from the distal nephron to compensate for the excretion of anions. However, none of the *in vitro *studies supported these concepts since not one of these studies reported studies of bone demineralization or any adverse effects (such as activation of osteoclasts or enzymes) within the physiological range (7.35 to 7.45).

In contrast, the response to an alkaline diet on systemic pH *in vivo *has been documented in a randomized study to be 0.014 pH units [14]. It has been proposed that the osteoclast cell secretes acid to dissolve mineral during the bone remodeling cycle [262] and it has been shown that osteoclast cells respond to changes in pH [103]. However, there is no evidence to support the suggestion that the very small systemic pH changes (< 0.02 pH units) seen in response to diet [14] or possibly in response to bicarbonate salt changes actually influence the bone demineralization activities of osteoclasts cells or that diet acid becomes concentrated in the bone milieu. Further work is required to determine whether this is the case. Claims that these cell culture studies support the acid ash hypothesis are not supported by the evidence.

#### Phosphate

According to the acid-ash hypothesis, excess dietary "acid" from phosphate causes increased urine skeletal calcium excretion and loss of calcium, and the main source of dietary "acid" is dietary phosphate [152]. Meta-analyses of randomized studies of phosphate supplements did not see either of the hypothesis predicted effects of increased urine calcium and decreased calcium balance, and thus did not support the hypothesis regarding the role of phosphate.

#### Protein

According to the acid-ash hypothesis, higher dietary protein intakes are detrimental to bone health since protein is an important acid generating diet component and bone mineral is dissolved to neutralize acids and avoid systemic acidosis [9,14-16]. Increased calcium in the urine has been considered confirmation of this theoretical effect [15,18-21]. However, randomized trials of the amount [57,62,67,69] and type (animal versus vegetable) [59] of protein, and a meta-analyses of superior methodology randomized cross-over studies of protein intakes on calcium balance [29] do not support this proposed negative relationship between higher protein intakes and negative bone calcium retention.

Further, protein has positive effects on BMD, based on a meta-analysis of protein supplementation RCTs on spine BMD [27] and randomized trials that assessed the effect of protein after a hip fracture on BMD [263,264]. Therefore the assertion that higher protein intakes lead to osteoporosis is not upheld by the current evidence.

#### Potassium

Although seven randomized intervention studies reported decreased BR markers of nine [60,68] to 28 [72] percent in response to potassium salt ingestion [58,60,65,68,70-72] the BR markers did not decrease by a sufficient magnitude [46,47] to be considered to have made an important effect on bone resorption. The prospective observational study by Tucker at al. that found an association between potassium intakes and fruit and vegetable intakes with less BMD loss did not control for weight loss during follow-up, family history of osteoporosis, baseline BMD [75], and thus their finding could have been confounded by any of these osteoporosis risk factors. The three other observational studies did not find an association between potassium intakes and better bone health outcomes [77,79,83]. Therefore, the evidence does not support the acid-ash hypothesis tenet that potassium is bone protective.

#### Protein and calcium interaction

Some of the writers have suggested that calcium and protein work in an interaction such that protein is moreso or only detrimental to bone health when calcium intakes are low. The evidence to substantiate this assertion was limited to this single observational study [81], while one randomized trial did not support the idea [69]. Since the observational study that supported the idea that protein was detrimental to bone health when calcium intakes were low did not control for weight loss during follow-up, baseline BMD, and vitamin D status their finding may have been due to uncontrolled confounding. The randomized trial that examined the effect of two levels of calcium intake with two levels of protein intake revealed that protein was not detrimental to calcium balance whether calcium intakes were high or low. Thus the concept that higher protein intakes are detrimental to bone health when calcium intakes are low was not supported by the evidence.

In terms of the biological plausibility, the acid-ash hypothesis is not supported by research evidence regarding a mechanism that functions at physiological pH or the plausible roles of phosphate, sodium, potassium, protein, and a protein-calcium interaction in bone health (Table 5).

### Hill's Criterion #5: Consistency

To evaluate Hill's criterion regarding consistency which demands consistent evidence from a variety of study design elements to support a causal relationship, we examined the prospective observational studies on the acid ash hypothesis. Since three [75,77,79] of the five prospective cohort studies that supported the hypothesis [73,75,77,79,81] also had some findings (regarding some posited acid or alkaline nutrients) that did not support the hypothesis [75,77,79], these studies did not demonstrate internal consistency in their support of the hypothesis. Further, several osteoporosis risk factors [49-52] were not controlled for in the hypothesis supporting cohort studies including: weight loss during follow-up [74,75,81], parental history of osteoporosis [73-75,79], baseline BMD [73-75,77,81], vitamin D status [74,75,77,79], and estrogen status [77] (Table 3). Due to the limitations of the observational study design and with the lack of control for important risk factors for osteoporosis, the prospective observational studies cannot clearly support a cause and effect relationship between diet derived acid and bone health. Thus, although quoted as proof of the acid-ash hypothesis, the prospective observational studies do not support the acid-ash hypothesis due to potential uncontrolled confounding by osteoporosis risk factors (Table 5).

### Hill's Criterion #6: Experiment

Hill's criterion regarding Experiment requires that actual experiments be conducted to determine whether the frequency of a disease is altered by an exposure [31]. To be able to claim causation, experimental evidence should demonstrate that the hypothesized exposure induces or prevents the disease under study. To our knowledge, neither of the direct measures of bone strength or osteoporosis (fragility fractures or bone strength as measured from bone biopsy samples) have been used as outcomes in randomized intervention trials of the acid-ash hypothesis.

#### Changes in Bone Mineral Density

Two randomized control trials have used changes of BMD as the outcome measure in post menopausal women with opposite findings [65,68]. A useful clinical measure of bone, BMD is not a direct measure of osteoporosis, but a surrogate measure of this disease. Changes of BMD are, therefore not ideal measures of osteoporosis. The two randomized control trials that measured changes of BMD as their outcome measures in studies of the acid ach hypothesis differed in quality by 2 important risks of Bias [43]: concealment of allocation and selective outcome reporting [65]. During the randomization process, when a study is designed to ensure that the randomization process is not tampered with, one can have more confidence that the study groups are equivalent in terms of known and unknown confounders. Concealing the allocation to the study groups by concealing the process, or making it unalterable by the investigators, is an indicator of study quality [43]. Thus the MacDonald et al study of potassium citrate and increased fruit and vegetable intakes on the change of BMD [65] was therefore considered the higher quality study. This study found no effect of the potassium citrate, or increased fruit and vegetable intakes on BMD over time [68], which is more likely to be an accurate reflection of the truth since it is less likely to be biased.

#### Bone resorption markers (BR marker)

The BR marker results did not support the acid-ash hypothesis. The inclusive analysis of the BR markers demonstrated significant heterogeneity by the more alkaline interventions, and thus suggests that the individual studies should not be combined. The finding of heterogeneity suggests that the various interventions (Table 1) to alter acid load, such as altering protein, fruit and vegetables intakes, or giving a bicarbonate or citrate salt are not equivalent, even though they are considered to be the same under the acid ash hypothesis. The sensitivity meta-analysis was sufficiently homogenous to proceed with meta-analysis, and the results revealed no significant difference in BR markers in response to a change in diet acid or alkaline load (p = 0.76). The results from the one individual study that demonstrated a clinically important difference in the BR marker measured in fasting urine found that N-telopeptide decreased in response to protein supplementation [58], a finding which is opposite direction to that which the hypothesis would predict. In summary, the BR marker results suggest no important changes in BR markers from an alkaline diet.

Thus, randomized controlled studies of the acid ash hypothesis using calcium balance, change of BMD, or BR markers do not support the hypothesis (Table 5).

## Discussion Overview

This systematic review, based on quality randomized trials and prospective observational studies, did not find support for the acid-ash hypothesis which states that "acid" from the modern diet causes osteoporosis or that an alkaline diet or "alkaline" supplements or salts prevent osteoporosis. Applying Hill's criteria to this body of evidence provides additional insight into the likelihood of causality based on established criteria. The criterion related to the strength of association partially was met because the quantity of urine calcium was related to the diet acid load, however, calcium balance which is the preferred measure of calcium metabolism, was not related to diet acid load. The criteria for the biological gradient, biologic plausibility, consistency and experimental evidence for a casual relationship for the acid ash hypothesis were lacking with regard to whole body calcium balance, bone resorption markers, and changes in BMD. Thus, claims that the modern "acid" producing diet causes osteoporosis were not substantiated by research evidence. These finding suggest there is not likely any bone health benefit from consumption of commercial products intended to counteract this dietary acid.

Hill's criterion requires consistent evidence from a variety of experimental designs and the studies of food estimates of acid load remain inconsistent and show only associations with urine calcium rather than a causal relationship for osteoporosis. Although there is general agreement in the commercial literature and advertising about which foods contribute acid and base, the foundation of these statements is weak and it is unclear whether the calculated amounts of acid or base have any association with health or disease.

In addressing these findings within the context of the existing acid-ash hypothesis, limitations to the evidence can be identified. Evidence or limitations to the evidence arise in the following areas: 1) lack of support for the hypothesis by well-designed calcium balance studies; 2) lack of well-designed studies with more direct measures of this disease (bone strength as measured by fragility fractures or biomechanical testing of bone biopsy material); 3) lack of control of important potential risk factors among the longitudinal cohort studies; and 4) lack of a defined mechanism that could occur at physiological pH. Small alterations in the surrogate measures of calcium in the urine and/or changes in BR markers are not evidence that alterations in the diet acid load cause bone demineralization. Additionally biological plausibility is questionable because of the conflicting roles of phosphate, sodium, potassium, protein and calcium interactions, and milk, since the roles put forth by the hypothesis differ from the actual roles of these molecules with respect to osteoporosis.

In the acid-ash model, sodium is one of the cations that has been assumed to represent base excretion, and cations theoretically protect against bone calcium losses [15]. In the model of the acid-ash hypothesis, sodium is considered to have a similar bone protective effect to calcium, potassium, and magnesium. However, experts consider high sodium intakes to be a possible risk factor for bone mineral loss [48]. It is possible that the acid-ash hypothesis is over simplistic in its categorization of sodium, potassium, calcium and magnesium as protective ions *vis-à-vis *bone health.

Early work to define food sources of acid and base began early in the previous century [110,112,265]. Sherman published tables listing the acid and base contributions of 64 foods based on the ashed foods' mineral content in 1912 [110]. Remer and Manz updated the original calculation in 1995 when they published newer tables of food acid loads with simple correction factors for each mineral designed to take imperfect absorption into account [152]. The 1912 and 1995 food lists share a premise that urinary excretion of hydroxides of sodium, potassium, calcium and magnesium reflect "base" excretion while urinary protonated forms of phosphate, sulfate, and chloride reflect "acid" excretion [110,152,265]. However, the assumption that food lists can reliably and exclusively classify foods as dietary sources of excreted acid or base is not supported by this review. The food lists categorize dietary phosphate and protein containing foods as acid sources anticipated to enhance bone loss, while evidence suggests that dietary phosphate does not increase calcium excretion (Figure 4) or decrease calcium balance (Figure 5), and dietary protein may enhance or protect BMD [263,264]. Although Remer and Manz estimated that milk has a slight acidic load [152], other investigators estimated that milk would supply an alkaline load [110,122], and a recent study revealed that milk actually contributes an alkaline load [207]. Grains were included in the food lists as acid generating, and have been considered "acid-yielding" [17], but have not been evaluated for their hypothesized acidogenic and calciuric responses or effect on bone health, although two attempts have been made [133,197].

The measurement of the diet acid load based on urine titratable acidity, ammonia and bicarbonate [14,15,115] is not a precise estimate due to problems with each constituent [24]. Ammonium (as ammonia) and bicarbonate (as CO_2_) may be lost due to volatility [266] prior to their measurement. Additionally, the measurement of titratable acidity is influenced by poor solubility of calcium, phosphorus, and uric acid, which can cause an over or underestimation of titratable acidity [24,267]. Therefore the measurement of urinary acid excretion is error prone and may not accurately reflect the exposure to dietary acidity.

The majority of experimental evidence supporting the acid-ash hypothesis is derived from studies that have used urine calcium and/or BR markers as the outcome measure. Urine calcium changes are confounded by changes in calcium absorption. The estimated change in BR markers (Figures 6 &7) is less than the "least significant change" needed to consider that a true biological effect has occurred as opposed to a change due to measurement error [46,47,268] and the results are inconsistent. The better-designed RCT which used changes of BMD as the outcome did not support the hypothesis [68]. The lack of consistent information regarding the effects of protein, milk and grain foods on bone undermines support for the acid-ash hypothesis, and the unreliable measurement of acid excretion in urine further undermines the hypothesis (Table 5). Therefore, the experimental evidence does not support the acid-ash hypothesis (Table 5).

The acid-ash hypothesis recommends that to maintain bone health people consume generous quantities of fruit and vegetables (8 to 10 servings per day [145,152,242]) along with modest amounts of grain and protein foods [21,42]. Generous quantities of fruit and vegetables are not likely to be harmful and may have other health benefits [269]. It is possible that fruit and vegetables are beneficial to bone health through mechanisms other than via the acid-ash hypothesis since there is some preliminary human and animal evidence that some fruits and some vegetables have supportive effects on bone [270,271].

In contrast to the acid-ash hypothesis, recent research suggests that sufficient protein intake is needed for the maintenance of bone integrity [27,29,62,263,263,264]. Changes in urine calcium and BR markers should not be considered proof of the acid-ash hypothesis.

### Strengths and Limitations

The primary strengths of this study are that we conducted a broad search of the literature, only included studies with randomized or prospective cohort study designs, and focused on the higher quality randomized studies, evaluated these studies for risk of bias, as recommended for systematic reviews [36]. We recognize the limitation of a systematic review is that the conclusions are based on the available studies. To formally reject the acid-ash hypothesis, well-designed unbiased studies with adequate rigor [34-36,36] are needed, using direct measures for osteoporosis bone fragility: biomechanical testing of bone or the incidence of fragility fractures [259,260].

## Conclusions

Based on the review of the literature to date and an application of Hill's criteria to the evidence the relationship between dietary acid with risk of osteoporosis is not confirmed. Further research is needed to determine whether fruit and/or vegetables are protective of bone health and what are the ideal protein intakes for bone health.

## Abbreviations

BMD: Bone mineral density; BR markers: bone resorption markers; CI: confidence interval; CTX: C-telopeptide; DPD: deoxypyridinoline; NAE: net acid excretion; RCT: randomized controlled trial;

## Competing interests

The authors declare that they have no competing interests.

## Authors' contributions

The author's responsibilities were as follows: TRF, SCT, & AWL designed the study, TRF, SCT, AWL & DAH extracted the data; TRF searched the literature, performed the statistical analysis and wrote the manuscript. ME directed the study's statistical analysis and graphic representations, SCT and TRF independently rated the randomized studies for their risk of bias using the Cochrane Risk of Bias Tool. All of the authors contributed to interpret the findings and writing the manuscript, and all authors read and approved the final manuscript.

## Funding

This work was supported in part by: Doctoral fellowships from the University of Calgary and the Alberta Heritage Fund for Medical Research. The funders played no role in study design, collection, analysis, interpretation of data, writing of the report, or the conclusions reached.
